# Effects of coronavirus disease 2019 in patients with optic neuritis: a single-centre retrospective cohort study

**DOI:** 10.1007/s10072-025-08365-7

**Published:** 2025-07-29

**Authors:** Rong Yan, Yanjun Guo, Chao Meng, Xiuyun Kong, Jiawei Wang

**Affiliations:** Department of Neurology, Beijing Tongren Hospital, Capital Medical University, Beijing, 100176 China

**Keywords:** Coronavirus disease 2019, Optic neuritis, Acute disseminated encephalomyelitis, AQP4-IgG, MOG-IgG

## Abstract

**Background:**

The relationship between coronavirus disease 2019 (COVID-19) and optic neuritis (ON) remains unclear, with limited evidence on its clinical features and outcomes. This study aimed to investigate the impact of COVID-19 on ON subtypes and prognosis.

**Methods:**

We conducted a single-centre retrospective cohort study, comparing acute ON patients with and without recent COVID-19 infection during the omicron wave (December 2022 to January 2023). A historical control group from the pre-COVID-19 era was included for comparison.

**Results:**

A total of 55 ON patients were included, of whom 12 had recent COVID-19 infection. COVID-19-associated ON showed a higher incidence of acute disseminated encephalomyelitis-associated ON (ADEM-ON, 25% vs. 0%) and a lower rate of aquaporin-4 antibody-associated ON (AQP4-ON, 0% vs. 31.4%). These patients presented more frequently with eye pain (75%) and respiratory symptoms (60%). At onset, 66.7% experienced severe visual loss (mean logMAR 1.10 ± 0.71), but most (83.3%) achieved good visual recovery (mean logMAR 0.08 ± 0.44) after immunotherapy. Only one relapse was observed during follow-up.

**Conclusions:**

COVID-19 may trigger distinct autoimmune processes in ON, particularly increasing ADEM-ON prevalence. Despite initial severe vision loss, outcomes are generally favourable with timely treatment. These findings expand understanding of neuro-ophthalmic complications related to COVID-19.

## Introduction

Severe acute respiratory syndrome coronavirus 2 (SARS-CoV-2) is a novel viral infection responsible for the coronavirus disease 2019 (COVID-19) pandemic. Although COVID-19 is no longer a public health emergency of international concern, with the new variants of SARS-CoV-2 emerging, it remains a public health threat. Neuro-ophthalmic and central nervous system (CNS) complications, such as optic neuritis (ON), papilledema, cranial neuropathies, Miller Fisher syndrome, ocular myasthenia gravis, encephalitis and CNS inflammatory demyelinating diseases, of COVID-19 have been increasingly recognised since the pandemic [1, 2]. A recent multicentre, retrospective observational study revealed that the diagnosis rate of ON increased from the pre-COVID to the pre-vaccine period compared with other neuro-ophthalmic conditions [3]. ON is commonly indicative of autoimmune neurological disorders, including multiple sclerosis, myelin oligodendrocyte glycoprotein antibody-associated disease (MOGAD), and neuromyelitis optica spectrum disorder (NMOSD). Although the cause of ON is protease activity, CNS autoimmunity is the primary concern. Cytokine storms mediated by interleukins (ILs) (IL-6 and IL-8), resulting from the over-activation of innate immune responses, play a crucial role in the pathogenesis of COVID-19 and other systemic inflammatory syndromes [4]. Moreover, activation of the innate and adaptive immune systems by COVID-19 is associated with the development of autoantibodies and the onset of autoimmune conditions [5]. Despite the increasing number of case reports and small case series describing ON in association with COVID-19, comprehensive cohort studies systematically comparing the clinical features, subtypes, and outcomes of COVID-19-associated ON versus non-COVID-19 ON remain scarce. Existing literature is limited by small sample sizes, heterogeneous diagnostic criteria, and a lack of longitudinal follow-up data, making it difficult to understand the true impact of SARS-CoV-2 infection on ON subtypes and prognosis. To address this gap, we conducted a retrospective cohort study to evaluate the clinical and immunological characteristics of ON patients with recent COVID-19 infection during the Omicron wave. By comparing these patients to a historical control group from the pre-COVID-19 era, we aimed to clarify the differences in ON subtypes, particularly the prevalence of ADEM-ON and AQP4-ON, and to assess visual outcomes following immunotherapy. Our findings provide novel insights into the potential autoimmune mechanisms triggered by SARS-CoV-2 and offer valuable evidence for the early recognition and management of COVID-19-associated ON.

## Methods

### Study design and participants

This was a single-centre, retrospective study. We reviewed all acute ON patients with and without COVID-19 at the Department of Neurology of our hospital during the omicron pandemic wave from 1 December 2022 to 31 January 2023. As the World Health Organization (WHO) reported the first cases of COVID-19 were recorded in late December 2019, we enrolled ON patients one year before that time (between 1 December 2018 and 31 January 2019 in the pre-COVID period) as ON without COVID-19 for comparison.

**Fig. 1:**
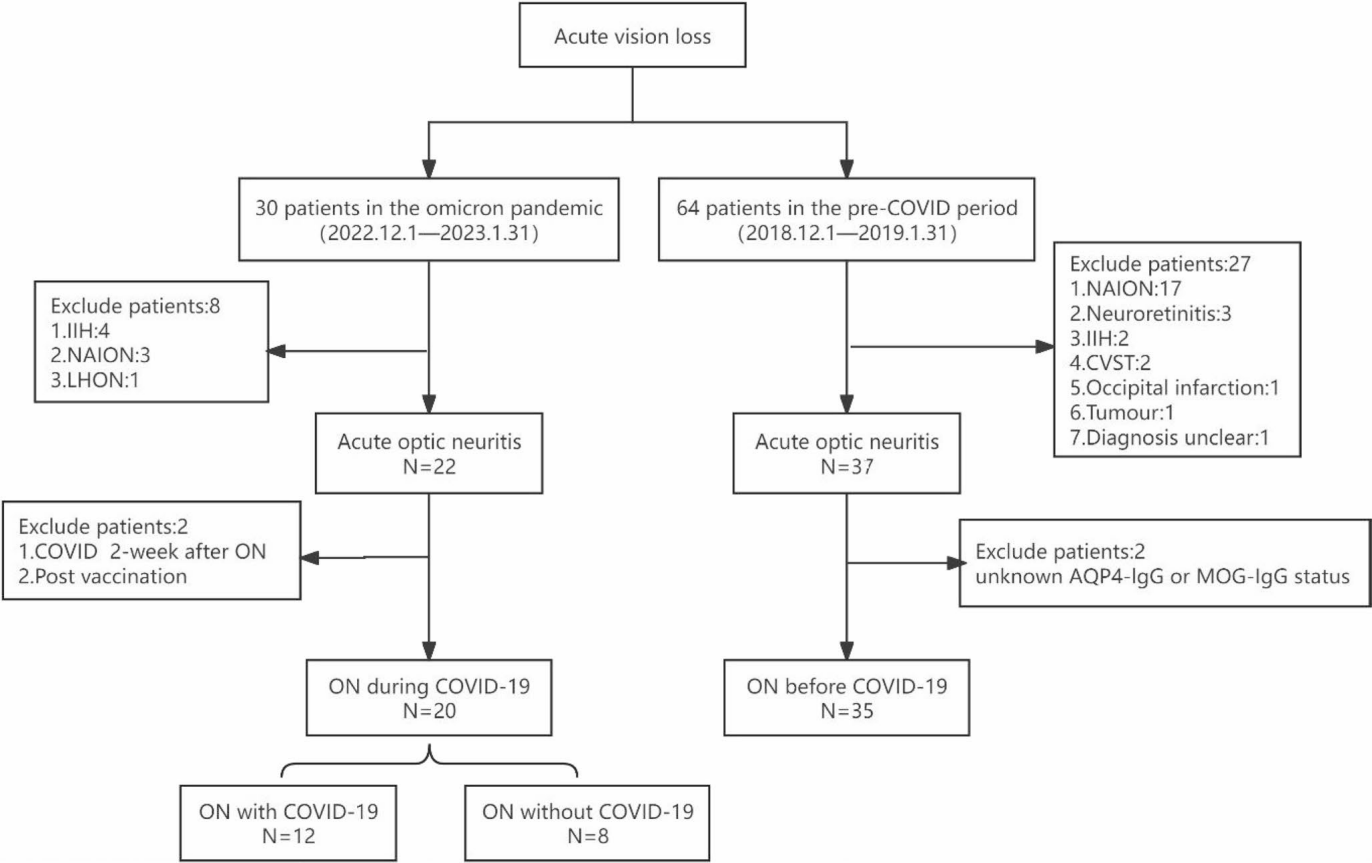
Flowchart of the enrolment process of patients with optic neuritis in the study cohort Respiratory infection*: respiratory infection within 6 weeks

ON subtypes were classified according to the latest diagnostic criteria: acute demyelinating encephalomyelitis associated ON (ADEM-ON), aquaporin 4 antibody associated ON (AQP4-ON), myelin oligodendrocyte glycoprotein antibody-associated ON (MOG-ON), multiple sclerosis-associated ON (MS-ON), collapsin response mediator protein 5 associated ON (CRMP5-ON), and other isolated ON (ION) [6]. COVID-19 met the World Health Organization COVID-19 case definitions or the “Novel Coronavirus Infection Diagnosis and Treatment Protocol (Trial Version 10)” issued by the National Health Commission of the People’s Republic of China in 2023. COVID-19-associated ON (ON with COVID-19) is defined as ON occurring within 6 weeks following the onset of symptomatic COVID-19 infection, based on either a positive reverse transcription polymerase chain reaction (RT-PCR) test or a positive antigen test result [7]. Patients who received a COVID-19 vaccine within the 6 weeks preceding ON onset were excluded from this category to minimize potential confounding factors related to post-vaccination immune responses. This study was approved by the Ethics Committee of Beijing Tongren Hospital (TRECKY2023-053), and the requirement for informed consent was waived because this was a retrospective case series. Figure 1 illustrates the flow chart for enrollment of patients with ON.

### Study methods

All patients included in our study underwent detailed neuro-ophthalmic and neurological examinations. Demographic information, clinical manifestations, blood and cerebrospinal fluid (CSF) laboratory results, optical coherence tomography abnormalities, magnetic resonance imaging (MRI) changes, treatments, and outcomes were collected from the electronic medical records of Beijing Tongren Hospital. AQP4-IgG and MOG-IgG were detected in all the patients using cell-based assays. CRMP5-IgG and GFAP-IgG (cell-based assays) were tested, if necessary. All ON patients with COVID-19 infection were followed up until September 2023. All the data were examined by at least two experienced neurological physicians.

### Visual recovery and relapse assessment

Best-corrected visual acuity (BCVA) was measured by Snellen decimal visual acuity (VA), with Snellen decimal VA conversion to logMAR VA as the resolution for analysis. Snellen decimal VA < 0.1 was defined as severe vision loss (SVL), whereas Snellen decimal VA ≥ 0.5 was defined as good recovery (GR). Relapse was defined as a neurologic deficit associated with an acute inflammatory demyelinating event that lasts at least 24 h in the absence of fever and infection, and the current attack must be separated from a prior attack by at least 30 days.

### Outcomes of interest

The primary outcome of interest in this study was the difference between subtype of ON with and without COVID-19. The secondary outcomes included VA recovery from ON with COVID-19, severity of COVID-19, and CNS complications. VA, subtype classification, and other clinical features were also compared between ON with and without COVID-19.

### Statistical analyses

Clinical data were summarized using descriptive statistics. Continuous variables were reported as mean ± standard deviation (SD), and categorical variables were reported as percentages *N* (%). Comparisons between the two groups were performed using the independent Student’s t-test for continuous variables including age and logMAR VA, and the chi-squared test or Fisher’s exact test for categorical variables. Statistical comparisons of the logMAR VA at nadir and the VA follow-ups were performed using the Wilcoxon signed-rank test. The significance level was set at *P* < 0.05. The Bonferroni correction was used to adjust for multiple comparisons. GraphPad Prism version 8 and SPSS version 26.0 were used for statistical analyses.

## Results

### Demographic and clinical features

The demographic and clinical details are summarized in Table 1. A total of 55 ON patients were included: 20 patients during the COVID-19 pandemic wave and 35 patients in the pre-COVID-19 era. Among 20 patients during the pandemic, 12 were confirmed to have COVID-19 infection, while 8 tested negative for SARS-CoV-2. Compared to ON cases in the pre-COVID-19 era, patients with ON during the pandemic period demonstrated a higher incidence of eye pain (80% vs. 51.4%, *P* = 0.036), respiratory infection (60% vs. 17.1%, *P* = 0.001) and ADEM-ON (15% vs. 0%, *P* = 0.043). There was no significant difference in other indicators such as age, gender, bilateral involved, history of ON between the two groups (*P* > 0.05).

**Table 1:** Demographic and clinical manifestations of optic neuritis between patients during and before COVID-19

Variables	All (*N* = 55)	ON during COVID-19 (*N* = 20)	ON during pre-COVID era (*N* = 35)	*P* value
Age(years)	40.56 ± 14.79	41.60 ± 14.14	39.97 ± 15.32	0.698
Gender (%)				> 0.999
Male	13 (23.6)	5 (25.0)	8 (22.9)	
Female	42 (76.4)	15 (75.0)	27 (77.1)	
Bilateral involved (%)	16 (29.1)	9 (45.0)	7 (20.0)	0.05
Eye pain(%)	34 (61.8)	16 (80.0)	18 (51.4)	**0.036**
Optic disc edema(%)	32 (58.2)	13 (65.0)	19 (54.3)	0.438
LogMAR VA at nadir	1.19 ± 0.72	1.24 ± 0.70	1.17 ± 0.74	0.756
History of ON(%)	23 (41.8)	7 (35.0)	16 (45.7)	0.438
Respiratory infection* (%)	18 (32.7)	12 (60.0)	6 (17.1)	**0.001**
MOG-IgG(%)	11 (20.0)	4 (20.0)	7 (20.0)	> 0.999
AQP4-IgG(%)	15 (27.3)	4 (20.0)	11 (31.4)	0.360
AIA(%)	22 (40)	9 (45.0)	13 (37.1)	0.567
Optic nerve MRI				
LEON(%)	27 (49.1)	11 (55.0)	16 (45.7)	0.508
chiasmal involvement(%)	6 (10.9)	4 (20.0)	2 (5.7)	0.175
perineural enhancement(%)	5 (9.1)	2 (10.0)	3 (8.6)	> 0.999
ON subtype				
ADEM-ON(%)	3 (5.5)	3 (15.0)	0 (0)	**0.043**
AQP4-ON(%)	15 (27.3)	4 (20.0)	11 (31.4)	0.360
MOG-ON(%)	11 (20.0)	4 (20.0)	7 (20.0)	> 0.999
MS-ON(%)	1 (1.8)	1 (5.0)	0 (0)	0.364
ION(%)	25 (45.5)	8 (40.0)	17 (48.6)	0.539

Data was expressed as mean ± SD or *n* (%). AIA: autoimmune antibodies, including antinuclear antibody, anti-double-stranded DNA, anti-Sm, Sjögren syndrome A or B, anticardiolipin (ACL and β2-GPI), antineutrophil cytoplasmic antibodies, rheumatoid factor, and human leucocyte antigen B27 phenotype

In addition, we subdivided patients during the COVID-19 pandemic wave into two groups: ON with confirmed COVID-19 infection (COVID-19 positive): ON onset within 6 weeks of laboratory-confirmed SARS-CoV-2 infection. ON without confirmed COVID-19 infection (COVID-19 negative): patients presenting with ON during the same period but without evidence of SARS-CoV-2 infection. For the pre-COVID-19 control group, patients were further stratified based on the presence or absence of upper respiratory tract infections within 6 weeks prior to ON onset. This classification was intended to assess whether respiratory infections, in general, contributed to the variation in ON subtypes and clinical features. During the COVID-19 period, ADEM-ON appeared more frequently among patients with confirmed infection compared to other subgroups (25% vs. 0%, *p* = 0.010). In contrast, ON patients without COVID-19 more often reported eye pain (*p* = 0.049), and in the pre-COVID cohort, those with recent respiratory infections showed a higher proportion of peripapillary optic disc edema (*p* = 0.012). Comprehensive subgroup data are summarized in Table 2.

**Table 2:** Clinical characters of optic neuritis with or without respiratory infection during COVID-19 and pre-COVID era

Variables	ON during COVID-19		ON during pre-COVID era		*P* value
	COVID-19 positive (*N* = 12)	COVID-19 negative (*N* = 8)	With URI (*N* = 6)	Without URI (*N* = 29)	
Age(years)	44.08 ± 14.09	37.88 ± 14.30	35 ± 7.61	41 ± 16.38	0.648
Gender (%)					0.925
Male	3(25)	2(25)	2(33.3)	6(21.7)	
Female	9(75)	6(75)	4(66.7)	23(79.3)	
Bilateral involved (%)	6(50)	3(37.5)	0	7(24.1)	0.131
Eye pain(%)	9(75)	7(87.5)	5(83.3)	13(44.8)	**0.049**
Optic disc edema(%)	10(83.3)	3(37.5)	6(100)	13(44.8)	**0.012**
LogMAR VA at nadir	1.10 ± 0.71	1.44 ± 0.68	0.88 ± 0.28	1.23 ± 0.79	0.757
History of ON(%)	5(41.7)	2(25)	0	16(55.2)	0.061
MOG-IgG(%)	4(33.3)	0	2(33.3)	5(17.2)	0.247
AQP4-IgG(%)	0	4(50)	2(33.3)	9(31.0)	0.075
AIA(%)	7(58.3)	2(25)	2(33.3)	11(37.9)	0.459
Optic nerve MRI					
LEON(%)	5(41.7)	6(75)	4(66.7)	12(41.4)	0.279
chiasmal involvement(%)	1(8.3)	3(37.5)	1(16.7)	1(3.4)	0.051
perineural enhancement(%)	2(16.7)	0	0	3(10.3)	0.515
ON subtype					
ADEM-ON(%)	3(25)	0	0	0	**0.010**
AQP4-ON(%)	0	4(50)	2(33.3)	9(31.0)	0.075
MOG-ON(%)	4(33.3)	0	2(33.3)	5(17.2)	0.247
MS-ON(%)	0	1(12.5)	0	0	0.112
ION(%)	5(41.7)	3(37.5)	2(10.9)	15(51.7)	0.780

URI, upper respiratory tract infections;

All patients with COVID-19 had laboratory evidence of COVID-19. Three patients tested positive for COVID-19 in reverse transcription polymerase chain reaction tests, and nine patients tested positive in a throat swab antigen test. Although viral strains were not routinely tested in all patients, based on epidemiological data from our locality during the study period, most COVID-19 cases were likely caused by the omicron variant of SARS-CoV-2. None of our patients with COVID-19 had serious manifestations: eight (66.7%) and four (33.3%) patients had mild and moderate symptoms, respectively. Two patients used antiviral drugs (nirmatelvir/ritonavir or azvudine), and the other patients relieved their symptoms with supportive medication. The onset of ON with COVID-19 was − 2 to 25 days after COVID-19 was diagnosed (average, 10.42 ± 7.1 days). The subtypes of ON with COVID-19 were as follows: four (33.3%) cases of MOGAD, three (25%) cases of ADEM-ON, and five (41.7%) cases of ION. Detailed clinical information of ON with COVID-19 are summarised in Tables 3 and 4.

**Table 3:** Baseline clinical information of ON with COVID-19

Case/sex/age	Involved eye	Time interval (days)	Eye pain	LogMAR VA at nadir	Optic disc edema	MOG-IgG	Past history
1/F/51	OS	18	Y	0.2/1.9	N	N	ADEM
2/M/60	OU	9	Y	1.9/1.9	Y	N	N
3/F/62	OU	6	N	1.9/1.9	Y	N	N
4/F/69	OS	4	Y	0/0.4	N	1:100	MOGAD
5/F/26	OU	13	Y	2.3/1.9	Y	1:32	N
6/M/28	OU	13	Y	0/1.2	Y	1:100	N
7/F/39	OU	14	Y	1.3/1	Y	1:100	N
8/F/39	OD	25	N	0.7/0.1	Y	N	N
9/M/29	OU	11	Y	1.6/1.3	Y	N	N
10/F/48	OD	-2	N	1.9/1.6	Y	N	ION
11/F/37	OD	10	Y	0.7/0.4	Y	N	ION
12/F/41	OS	4	Y	0/0.3	Y	N	ION

Time interval: Time interval from COVID-19 symptom onset to optic neuritis

**Table 4:** Test information and interventions of ON with COVID-19

Case/sex/age	Neurologic manifestations	Optic nerve MRI	CSF	ON subtype	Treatment
1/F/51	Left limb numbness	Left intracanalicular enhancement	WBC, protein↑ OB negtive	ADEM-ON	IMVP + IVIG nirmatelvir/ritonavir
2/M/60	Numbness and weakness of both lower limbs, urinary retention	Bilateral intracanalicular lesion without enhancement	IgG↑ OB negtive	ADEM-ON	IMVP + IVIG azvudine
3/F/62	Left limb numbness, right facial paralysis	Bilateral intraorbital enhancement	IL-5,8↑ OB negtive	ADEM-ON	IVMP
4/F/69	N	Left intraorbital and intracanalicular enhancement with optic nerve sheath involvement	NA	MOG-ON	Oral prednisolone mycophenolate mofetil
5/F/26	N	Bilateral whole optic nerve enhancement with chiasmal involvement	IL-5,6,8↑	MOG-ON	IVMP
6/M/28	N	Bilateral intraorbital lesion without enhancement	IL-8↑	MOG-ON	IVMP
7/F/39	N	Bilateral intraorbital and intracanalicular enhancement	IL-8↑	MOG-ON	IVMP
8/F/39	N	Right intraorbital and intracanalicular enhancement	NA	ION	IVMP
9/M/29	N	Bilateral intraorbital enhancement	Normal	ION	IVMP
10/F/48	N	Left intraorbital lesion without enhancement	IL-8↑	ION	Oral prednisolone
11/F/37	N	Right intraocular enhancement with optic nerve sheath involvement	Normal	ION	IVMP
12/F/41	N	Left intraorbital and intracanalicular enhancement	IL-8↑	ION	IVMP

IVIG: Intravenous immunoglobulin, 0.4 g per kg body weight for five consecutive days

IMVP: Intravenous methylprednisolone, 500–1,000 mg/d for three to five consecutive days and continuous dose of oral prednisolone (1 mg/kg) gradually decreased

The distribution of subtypes of ON had a statistically significant difference between patients with and without COVID-19 (χ²=68.18, *P*<0.0001). ADEM-ON was observed more frequently in patients with confirmed COVID-19 infection (25%) compared to both COVID-19 negative and pre-COVID-19 cases (0%), based on the subtype distribution in our cohort. Conversely, AQP4-ON was absent in the COVID-19 positive group but present in 31.4% of pre-COVID-19 patients (*P* = 0.075).

### Imaging findings

**Fig. 2:**
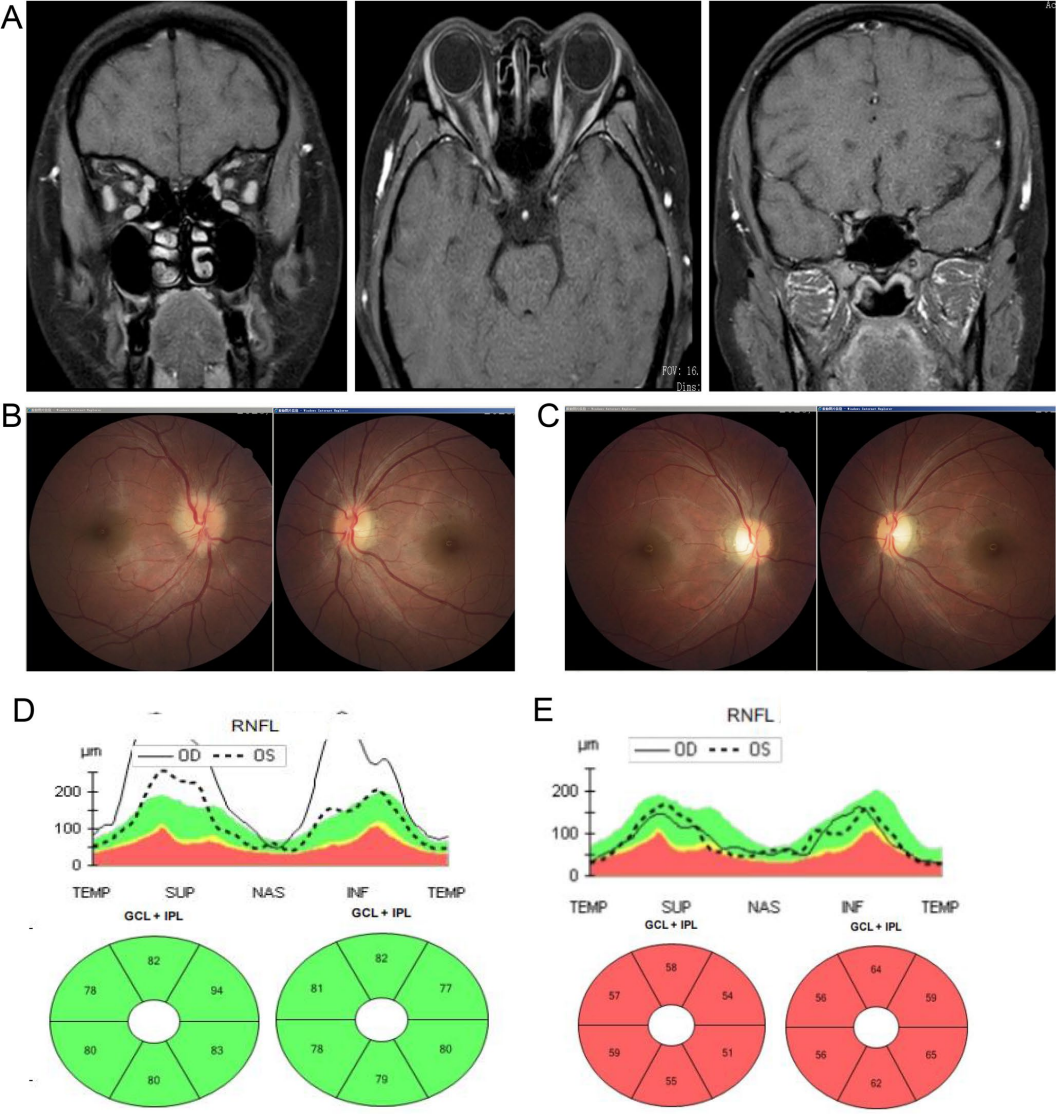
A 26-year-old woman without a significant past medical history presented with acute blurred vision of her both eyes with eye pain for 10 days during pregnancy (19 weeks). Thirteen days prior, she and her husband tested positive for coronavirus disease 2019 antigen after developing a fever, generalised body ache, and cough. The patient was serum myelin oligodendrocyte glycoprotein-immunoglobulin G-positive (1:32). (**A**). Optic nerve magnetic resonance imaging revealed the following: bilateral optic nerve orbital and canal segments, right optic nerve intracranial segment, and right chiasmal enhancement. (**B**). Bilateral optic disc oedema with Snellen decimal VA of HM/0.01 before treatment. (**C**) Bilateral optic disc pallor with Snellen decimal VA of 0.9/0.9 after 1.5 months. Optical coherence tomography revealed the following: a swollen retinal nerve fibre layer (RNFL) around the optic disc at initial presentation with normal ganglion cell layer (GCL) and inner plexiform layer (IPL) (**D**); 1.5 months after treatment, RNFL restored to normal with GCL and IPL thinning (**E**)

**Fig. 3:**
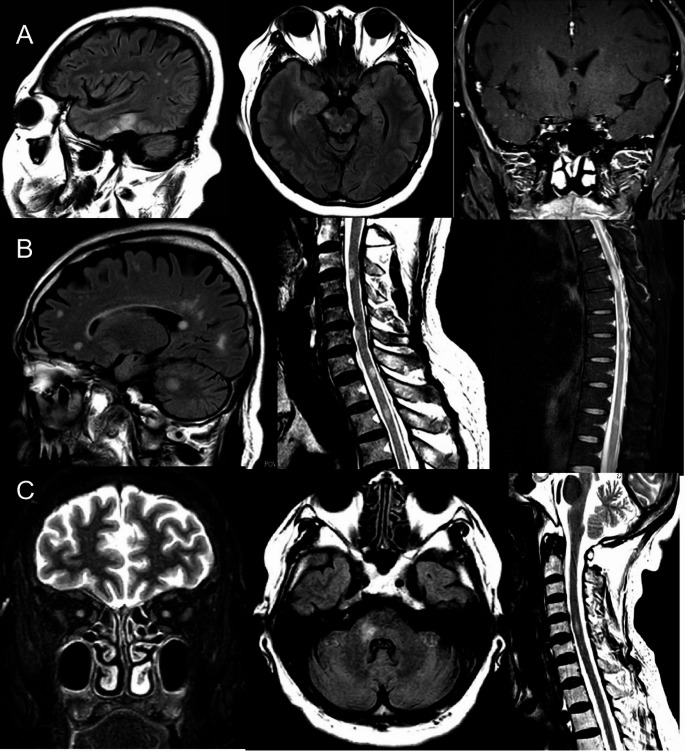
Three patients with acute disseminated encephalomyelitis-optic neuritis (ADEM-ON) and coronavirus disease 2019. All these patients were oligoclonal band-negative in the cerebrospinal fluid. Patient A was CRMP5-IgG-seropositive (titre 1:10) without cancer, was followed up for < 2 years, and had a good response to intravenous methylprednisolone, which did not meet the 2021 updated diagnostic criteria for paraneoplastic neurological syndrome at present. Patients B and C were both AQP4-IgG and MOG-IgG negative

All patients with COVID-19 had MRI abnormalities along the optic nerve: nine (75%) patients had optic nerve enhancement, five (41.7%) patients had long segments of the optic nerve involvement, and two (16.7%) patients had perineural enhancement (optic nerve sheath). The anterior segments of the optic nerve were predominantly involved (11 [91.7%] patients), with most involvement of the intra-orbital segments (nine [75%] patients), whereas one (8.3%) patient had longitudinally extensive optic nerve lesions with chiasmal involvement (Fig. 2). Six (50%) patients had brain MRI abnormalities, and three (3 [6.50%]) patients had ADEM-like lesions in the brain and spinal cord (Fig. 3). In the ON without COVID-19, 40 (93%) patients had MRI abnormalities along the optic nerve. 35(83.3%) patients had optic nerve enhancement, 22 (51.2%) had long segments of the optic nerve involvement, three (7%) had perineural enhancement (optic nerve sheath), five (11.6%) had chiasmal involvement and none had ADEM-like lesions on brain MRI.

### Laboratory findings

In COVID-19-associated ON, all serum samples were negative for AQP4 and GFAP antibodies, four (33.3%) patients were MOG-IgG-positive (titres were 1:100 in three patients and 1:32 in one patient), and one patient was CRMP5-IgG-positive (titre, 1:10). Seven (58.3%) patients had abnormal serum autoimmune antibody levels. Lumbar puncture was conducted in 11 patients, with an elevation of protein level in one patient, whereas CSF pressure, white blood cells, and oligoclonal bands were normal. An elevation of IL-8 levels was observed (7/9 [77.8%]), along with elevated levels of IL-5 and IL-6, whereas IL-10 and tumour necrosis factor-alpha levels were normal. In ON without COVID-19, seven (16.3%) patients were MOG-IgG-positive, 16(37.2%) were AQP4-IgG-positive, and the remaining patients were both negative. 15 (34.9%) patients showed abnormal serum autoimmune antibody levels.

### Treatment and clinical outcomes

**Fig. 4:**
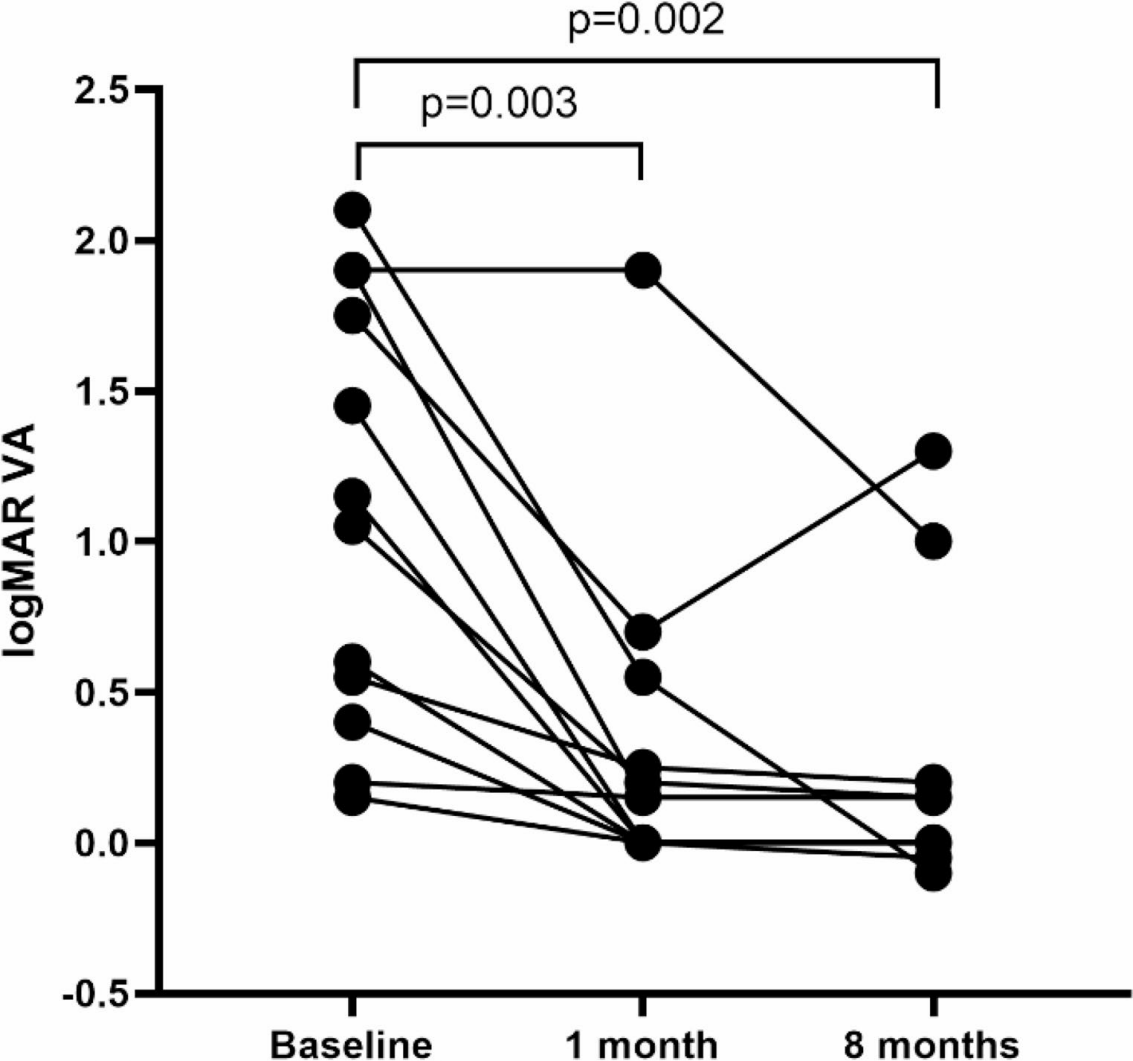
Comparison of the logMAR VA at the nadir and follow-up in patients with coronavirus disease 2019

In COVID-19-associated ON, all patients received intravenous methylprednisolone (IVMP) or oral prednisolone, and two patients with ADEM received intravenous immunoglobulin. All patients were followed up at least twice, and the median time of the last follow-up was 8 (0.75) months. At the end of the first month, the average logMAR VA was 0.15 ± 0.55; nine (75%) patients had GR, and one (8.3%) patient had SVL. At the end of the 8th month, the average logMAR VA was 0.08 ± 0.44; 10 (83.3%) patients had GR, and one (8.3%) patient relapsed during steroid tapering. The average LogMAR VA significantly improved compared with the baseline (*P* = 0.003, *P* = 0.002) (Fig. 4).

## Discussion

This study aimed to evaluate the impact of COVID-19 infection on patients with ON during the Omicron outbreak between December 2022 and January 2023. We observed several notable features in COVID-19-associated ON: (1) the proportion of acute disseminated encephalomyelitis-associated ON (ADEM-ON) was significantly higher in the COVID-19 group (25%) compared to 0% in the non-COVID groups; (2) COVID-19-associated ON typically developed approximately 10 days after infection, often presenting with severe visual impairment at onset, but most patients responded well to immunotherapy; (3) AQP4-IgG was not detected in the COVID-19-associated ON group, whereas the seropositivity rate was 31.4% in the pre-COVID group, suggesting a potentially meaningful absence of AQP4-ON in the context of COVID-19.

A synthesis of currently reported COVID-19-associated ON cases reveals several consistent trends [8–28]: we found that MOG-ON was the most common subtype among COVID-19-associated ON cases, while AQP4-ON remained rare and the majority of patients had favorable outcomes. In our review of 35 published COVID-19-associated ON cases (Table 5), MOGAD accounted for over half (51.4%), whereas only one case (2.86%) was identified as AQP4-ON. These trends align with our own findings in the COVID-19-positive ON subgroup, where MOG-ON accounted for 33.3% (4/12) and no cases of AQP4-ON were observed. Although the difference in AQP4-ON prevalence between the COVID-19-positive and pre-COVID groups did not reach statistical significance (*P* = 0.075), we cautiously interpret this as a potential trend rather than a definitive conclusion.

**Table 5:** Published cases of optic neuritis with COVID-19

Year	Literature	Age	Sex	Interval	COVID-19	Subtypes of ON	GR
2020	Sawalha et al. [8]	44	M	14	Mild	MOGAD	Yes
2020	Zhou et al. [9]	26	M	0	Mild	MOGAD	Yes
2020	Benito-Pascual et al. [10]	60	F	0	Moderate	ION+uveitis	Yes
2020	Catharino et al. [11]	64	M	0	Mild	ION	No
2020	de Ruijter et al. [12]	15	M	14	Asymptomatic	MOGAD	Yes
2020	Woodhall et al. [13]	39	F	0	Mild	MOGAD	No
2021	Žorić et al. [14]	63	M	28	Moderate	MOGAD	Yes
2021	Rodríguez-Rodríguez et al. [15]	55	F	0	Moderate	ION	No
2021	Kogure et al. [16]	47	M	0	Asymptomatic	MOGAD	Yes
2021	Sardar et al. [17]	38	F	14	Mild	ION	No
2021	Al-Salihi et al. [18]	33	F	7	Mild	ION	Yes
2021	Deane et al. [19]	21	F	7	Mild	ION	No
2021	Azab et al. [20]	32	M	10	Severe	ION	Yes
2021	Rojas-Correa et al. [21]	69	M	14	Mild	MOGAD	Yes
2022	Jossy et al. [22]	16	M	14	Mild	ION	Yes
		35	M	180	Mild	ION	No
		38	M	42	Mild	MOGAD	Yes
2022	Assavapongpaiboon et al. [23]	35	M	6	Mild	MOGAD	Yes
2022	Ide et al. [24]	34	F	14	Moderate	MOGAD + ADEM	Yes
2022	Kivanany et al. [25]	35	F	52	Moderate	AQP4-ON	No
2022	Tsouris et al. [26]	59	F	40	Mild	MOGAD + ADEM	Yes
2023	Bosello et al. [27]	74	F	60	Mild	MOGAD	Yes
2023^*^	Li et al. [28]	40.77 ± 9.87	F (61.5%)	19	Mild	MOGAD (46.2%)	Yes (92.3%)

2023 Li et al.^*^: In a case series of 13 patients (five males and eight females), six (46.2%) had myelin oligodendrocyte glycoprotein-optic neuritis (ON), whereas none had aquaporin-4-ON. Twelve (92.3%) patients had good recovery, excluding one patient

Notably, ADEM-ON was observed more frequently in the COVID-19-positive group (3/12), while absent in all other subgroups. Although this observation is limited by the small sample size, it suggests that SARS-CoV-2 infection may be associated with subtype-specific immune responses. Post-infectious demyelination triggered by molecular mimicry and innate immune activation may underlie this pattern. Supporting this, CSF analysis in several COVID-19-associated ON patients revealed elevated levels of IL-5, IL-6, and IL-8, indicating enhanced innate immune activity. However, as cytokine data were not available for non-COVID groups, these findings are presented as preliminary observations.

Our cohort also included several atypical cases. One pregnant patient presented with extensive optic nerve and chiasmal involvement on MRI, suggestive of AQP4-ON; however, she was seronegative for AQP4-IgG, positive for MOG-IgG at a low titer (1:32), and responded well to corticosteroids, supporting a diagnosis of MOGAD. Another patient with ADEM-ON tested positive for CRMP5-IgG at a low titer (1:10) and had no evidence of malignancy; follow-up is ongoing to evaluate the possibility of paraneoplastic neurological syndrome [29]. In one case, a patient experienced ON recurrence two days before testing positive for SARS-CoV-2 antigen, followed by fever and respiratory symptoms. Given the lag in antigen detection relative to infection onset, we considered this episode to fall within the spectrum of COVID-19-associated ON [30].

Taken together, our findings suggest that COVID-19 may be associated with shifts in the distribution of ON subtypes—particularly an increased occurrence of ADEM-ON and absence of AQP4-ON—potentially reflecting a distinct autoimmune mechanism from that of classical NMOSD. While the sample size is limited, these observations provide preliminary insights into the immunological profile of COVID-19-associated ON and warrant further validation in larger, prospective studies.

Although only one relapse was observed in the COVID-19 group during follow-up, the median follow-up duration of 8 months may not be sufficient to capture the long-term recurrence patterns of MOGAD or AQP4-ON. Longer-term surveillance is needed to better understand the disease course.

This study has several limitations. First, its single-center retrospective design may introduce selection and recall biases. Second, the small sample size, particularly in the COVID-19-positive subgroup, limits the statistical power for between-group comparisons. Third, visual outcome analysis was restricted to the COVID-19 group due to inconsistent follow-up durations and data completeness across other groups, precluding direct comparison. Fourth, although all patients in the COVID-19-negative group during the Omicron period tested negative by RT-PCR or antigen and reported no symptoms, asymptomatic or undetected infections could not be entirely excluded, potentially leading to misclassification bias. Lastly, viral genotyping was not performed, preventing analysis of potential associations between specific SARS-CoV-2 variants and ON subtypes.

## Conclusions

In this retrospective cohort study, COVID-19-associated ON was characterized by a higher incidence of ADEM-ON and lower AQP4-ON compared to pre-pandemic cases. Although severe vision loss was common at onset, most patients achieved good visual recovery following immunotherapy. These findings suggest that SARS-CoV-2 infection may be linked to distinct autoimmune demyelinating mechanisms in ON, particularly reflected in the increased frequency of ADEM-ON. By systematically outlining the clinical and immunological features of COVID-19-associated ON, this study provides meaningful implications for early identification and targeted management.

## Data Availability

The data used to support the findings of this study are available from the corresponding author upon request.

## Abbreviations

ADEM-ON: acute demyelinating encephalomyelitis associated ON
AQP4-ON: aquaporin 4 antibody associated ON
MOG-ON: myelin oligodendrocyte glycoprotein antibody-associated ON
MS-ON: multiple sclerosis associated ON
CRMP5-ON: collapsin response mediator protein 5 associated ON
ION: isolated ON
IIH: idiopathic intracranial hypertension
NAION: non-arteritic anterior ischaemic optic neuropathy
LHON: Leber hereditary optic neuropathy
CVST: cerebral venous sinus thrombosis
AIA: autoimmune antibodies
LEON: longitudinally extensive optic neuritis

## References

[1] Tisdale AK, Dinkin M, Chwalisz BK (2021) Afferent and efferent Neuro-Ophthalmic complications of coronavirus disease 19. J Neuroophthalmol 41(2):154–165 33935220 10.1097/WNO.0000000000001276

[2] Li Z, Lin D, Xu X et al (2023) Central nervous system complications in SARS-CoV-2-infected patients. J Neurol 270(10):4617–4631 37573554 10.1007/s00415-023-11912-x PMC10511589

[3] Zhao D, Li X, Carey AR, Henderson AD, Sight Outcomes Research Collaborative C (2024) Optic neuritis and cranial neuropathies diagnosis rates before coronavirus disease 2019, in the initial pandemic phase, and Post-Vaccine introduction. Ophthalmology 131(1):78–86 37634758 10.1016/j.ophtha.2023.08.021

[4] Alomari SO, Abou-Mrad Z, Bydon A (2020) COVID-19 and the central nervous system. Clin Neurol Neurosurg 198:106116 32828027 10.1016/j.clineuro.2020.106116 PMC7402113

[5] Gracia-Ramos AE, Martin-Nares E, Hernandez-Molina G (2021) New onset of autoimmune diseases following COVID-19 diagnosis. Cells;10(12) 10.3390/cells10123592 PMC8700122 34944099

[6] Petzold A, Fraser CL, Abegg M et al (2022) Diagnosis and classification of optic neuritis. Lancet Neurol 21(12):1120–1134 36179757 10.1016/S1474-4422(22)00200-9

[7] Ellul MA, Benjamin L, Singh B et al (2020) Neurological associations of COVID-19. Lancet Neurol 19(9):767–783 32622375 10.1016/S1474-4422(20)30221-0 PMC7332267

[8] Sawalha K, Adeodokun S, Kamoga GR (2020) COVID-19-Induced acute bilateral optic neuritis. J Investig Med High Impact Case Rep 8:2324709620976018 33238757 10.1177/2324709620976018 PMC7705770

[9] Zhou S, Jones-Lopez EC, Soneji DJ, Azevedo CJ, Patel VR (2020) Myelin oligodendrocyte glycoprotein Antibody-Associated optic neuritis and myelitis in COVID-19. J Neuroophthalmol 40(3):398–402 32604245 10.1097/WNO.0000000000001049 PMC7382408

[10] Benito-Pascual B, Gegundez JA, Diaz-Valle D et al (2020) Panuveitis and optic neuritis as a possible initial presentation of the novel coronavirus disease 2019 (COVID-19). Ocul Immunol Inflamm 28(6):922–925 32870739 10.1080/09273948.2020.1792512

[11] Catharino A, Neves MAO, Nunes N et al (2020) COVID-19 related optic neuritis: case report. J Clin Neurol Neurosci 1:10

[12] de Ruijter NS, Kramer G, Gons RAR, Hengstman GJD (2020) Neuromyelitis Optica spectrum disorder after presumed coronavirus (COVID-19) infection: A case report. Mult Scler Relat Disord 46:102474 32892062 10.1016/j.msard.2020.102474 PMC7462544

[13] Woodhall M, Mitchell JW, Gibbons E, Healy S, Waters P, Huda S (2020) Case report: Myelin oligodendrocyte glycoprotein Antibody-Associated relapse with COVID-19. Front Neurol 11:598531 33324337 10.3389/fneur.2020.598531 PMC7724101

[14] Zoric L, Rajovic-Mrkic I, Colak E, Miric D, Kisic B (2021) Optic neuritis in a patient with seropositive Myelin oligodendrocyte glycoprotein antibody during the Post-COVID-19 period. Int Med Case Rep J 14:349–355 34079389 10.2147/IMCRJ.S315103 PMC8165557

[15] Rodriguez-Rodriguez MS, Romero-Castro RM, Alvarado-de la Barrera C, Gonzalez-Cannata MG, Garcia-Morales AK, Avila-Rios S (2021) Optic neuritis following SARS-CoV-2 infection. J Neurovirol 27(2):359–363 33755923 10.1007/s13365-021-00959-z PMC7986141

[16] Kogure C, Kikushima W, Fukuda Y et al (2021) Myelin oligodendrocyte glycoprotein antibody-associated optic neuritis in a COVID-19 patient: A case report. Med (Baltim) 100(19):e25865 10.1097/MD.0000000000025865 PMC8133173 34106635

[17] Sardar S, Safan A, Okar L, Sadik N, Adeli G (2021) The diagnostic dilemma of bilateral optic neuritis and idiopathic intracranial hypertension coexistence in a patient with recent COVID-19 infection. Clin Case Rep 9(6):e04347 34136250 10.1002/ccr3.4347 PMC8190579

[18] Al-Salihi MM, Rahman MM, Al-Jebur MS et al (2021) Optic neuritis concomitant with pituitary Macroadenoma in a patient with active COVID-19 infection: A case report. Int J Surg Open 35:100390 34568623 10.1016/j.ijso.2021.100390 PMC8384762

[19] Deane K, Sarfraz A, Sarfraz Z, Valentine D, Idowu AR, Sanchez V (2021) Unilateral optic neuritis associated with SARS-CoV-2 infection: A rare complication. Am J Case Rep 22:e931665 34120138 10.12659/AJCR.931665 PMC8212842

[20] Azab MA, Hasaneen SF, Hanifa H, Azzam AY (2021) Optic neuritis post-COVID-19 infection. A case report with meta-analysis. Interdiscip Neurosurg 26:101320 34312592 10.1016/j.inat.2021.101320 PMC8295047

[21] Rojas-Correa DX, Reche-Sainz JA, Insausti-Garcia A, Calleja-Garcia C, Ferro-Osuna M (2022) Post COVID-19 Myelin oligodendrocyte glycoprotein Antibody-Associated optic neuritis. Neuroophthalmology 46(2):115–121 35273416 10.1080/01658107.2021.1916044 PMC8903772

[22] Jossy A, Jacob N, Sarkar S, Gokhale T, Kaliaperumal S, Deb AK (2022) COVID-19-associated optic neuritis - A case series and review of literature. Indian J Ophthalmol 70(1):310–316 34937266 10.4103/ijo.IJO_2235_21 PMC8917537

[23] Assavapongpaiboon B, Apinyawasisuk S, Jariyakosol S (2022) Myelin oligodendrocyte glycoprotein antibody-associated optic neuritis with COVID-19 infection: A case report and literature review. Am J Ophthalmol Case Rep 26:101491 35313470 10.1016/j.ajoc.2022.101491 PMC8928700

[24] Ide T, Kawanami T, Eriguchi M, Hara H (2022) SARS-CoV-2-related Myelin oligodendrocyte glycoprotein Antibody-associated disease: A case report and literature review. Intern Med 61(8):1253–1258 35135920 10.2169/internalmedicine.8709-21 PMC9107978

[25] Kivanany PB, Raviskanthan S, Mortensen PW, Lee AG (2022) Antiaquaporin 4-Related optic neuritis and myelitis Post-COVID-19 infection. J Neuroophthalmol 42(4):e571–e573 34629397 10.1097/WNO.0000000000001347

[26] Tsouris Z, Provatas A, Bakirtzis C et al (2022) Anti-MOG positive bilateral optic neuritis and brainstem encephalitis secondary to COVID-19 infection: A case report. Neurol Int 14(4):991–996 36548183 10.3390/neurolint14040078 PMC9782579

[27] Bosello F, Marastoni D, Pizzini FB et al (2023) Atypical Myelin oligodendrocyte glycoprotein antibody-associated optic neuritis and acute demyelinating polyneuropathy after SARS-CoV-2 infection: case report and literature review. J Neuroimmunol 375:578011 36621074 10.1016/j.jneuroim.2022.578011 PMC9779985

[28] Li Y, Sun M, Xu X et al (2024) Clinical features and prognosis in the first episode of optic neuritis with COVID-19. Acta Ophthalmol 102(3):e398–e401 37823443 10.1111/aos.15789

[29] Graus F, Vogrig A, Muñiz-Castrillo S et al (2021) Updated diagnostic criteria for paraneoplastic neurologic syndromes. Neurology(R) Neuroimmunol Neuroinflammation;8(4) 10.1212/NXI.0000000000001014 PMC8237398 34006622

[30] Spudich S, Nath A (2022) Nervous system consequences of COVID-19. Science 375(6578):267–269 35050660 10.1126/science.abm2052

